# The Impact of Different Types of Abuse on Depression

**DOI:** 10.1155/2021/6654503

**Published:** 2021-04-13

**Authors:** Milen L. Radell, Eid G. Abo Hamza, Wid H. Daghustani, Asma Perveen, Ahmed A. Moustafa

**Affiliations:** ^1^Department of Psychology, Niagara University, Lewiston, NY, USA; ^2^Mental Health Department, Faculty of Education, Tanta University, Egypt; ^3^Department of Learning and Developmental Disabilities, Arabian Gulf University, Bahrain; ^4^Department of Psychology and Counseling, Faculty of Human Development, Sultan Idris Education University, Tanjong Malim, Perak Darul Ridzuan, Malaysia; ^5^School of Psychology & Marcs Institute for Brain, Behaviour and Development, Western Sydney University, Sydney, New South Wales, Australia; ^6^Department of Human Anatomy and Physiology, The Faculty of Health Sciences, University of Johannesburg, South Africa

## Abstract

Despite a large amount of research on depression and abuse, there is still a controversy on how abuse is measured and on childhood trauma's effect on the physiological function of adults. Here, we attempt to clarify the relationship between different types of abuse and depression while focusing on childhood abuse. This article, unlike prior research, provides an overview that addresses physical, psychological, and sexual abuse and their psychological impact on the victims. Results show that abuse is a vulnerability factor for a variety of mental and physical health problems and that psychological abuse is strongly associated with depression. More research is needed to understand (a) the role of abuse in the development and maintenance of depression and, in particular, longitudinal studies that also account for the large number of risk and protective factors that influence this relationship and (b) how different types of abuse can influence response to treatment among survivors with depression, in order to provide effective trauma-focused approaches to manage depressive symptoms.

## 1. Introduction

Abuse is a common issue in all societies around the world and can be defined as an action that intentionally harms, injures, or impairs an individual physically, psychologically, or socially [1]. Abuse may take different forms such as physical, sexual, and emotional as well as commercial exploitation. This could lead to either potential or actual harm to the individual's life, with an enormous effect on their mental and physical health, their dignity, and their future relationships [2]. Abuse can happen at any time and to anyone and has serious, long-lasting negative emotional, mental, and physical effects on the victim. These include an increased risk for psychiatric disorders, including depression [3]. Depression, otherwise known as major depressive disorder or clinical depression, is a common and a serious mood disorder. Individuals who suffer from depression experience persistent feelings of sadness and hopelessness, and they lose interest in the activities they once enjoyed [4, 5]. In addition to the emotional problems caused by depression, individuals can also present with physical symptoms, such as chronic pain or headaches [6]. To understand the etiology of the depression associated with abuse, it is critical to address the patient's history of abuse and the emotional pain it may cause [7].

The onset, progression, and prognosis of major depressive disorder are affected by genetic, environmental, and dispositional factors [8]. For example, both general life stressors, and those specifically associated with abuse, have long been considered leading contributors to the onset of depression [9], in which severe depression is strongly related to emotional and physical abuse [10]. This relationship can be explained by the fact that abuse affects growth, personality, cognition, and behavior and can increase the sensitivity to life stress, both in childhood and adulthood [11]. For example, abuse alters how an individual perceives themselves, as well as the world around them, with a far-reaching impact on self-esteem, physical and mental development, and social emotions [12, 13]. Furthermore, it is associated with increases in pessimistic thinking and negativity, feelings of sadness, and social avoidance and isolation [14]. As such, both short- and long-term physical abuse can significantly affect the whole trajectory of a person's life, to feel worthless, hopeless, helpless, and the loss of pleasure in daily activities as well as indulgence in self-suffering—all common symptoms of depression [15, 16]. All of these factors can be expected to have long-term effects on personality states and traits, some of which have been identified as predictors of the severity of depression [17]. In addition, childhood maltreatment is associated with more severe and recurrent depression, as well as treatment resistance [18]. This article will review research on the relationship between abuse and depression, with a focus on childhood abuse and its impact on the victim.

To conduct this literature review, we performed a Google Scholar search using different combinations of terms, such as physical, sexual, psychological, emotional, and childhood abuse, adversity, corporal punishment, and depression and its various synonyms, as well as stress and vulnerability, to locate peer-reviewed empirical studies, reviews, and meta-analyses that dealt with the relationship between abuse and depression. This database was selected because it is inclusive of both the social sciences and biomedical literature. We also conducted forward and backward searches from the most relevant search results, namely, those that specifically address the relationship between different types of abuse and depression. While there are large literatures dealing with abuse or depression alone, our main focus was on more recent studies, published within the last 5-10 years, that, ideally, compared the impact of different types of abuse on depression. This type of study, however, was rare, and we also included research that had considered only one type of abuse, with a focus on abuse that had occurred during childhood, and its relationship to depression. Beyond this, the review was not systematic. The lack of other formal exclusion criteria for the research we located, other than that it had to be peer reviewed, could be considered a limitation of this review.

## 2. Challenges in Abuse Research

One of the main challenges in reviewing the relationship between abuse and depression is the equivocal literature on the definition and the measurement of abuse [1, 19]. Although there is a clear distinction between three major types of abuse—physical, sexual, and psychological, they are assessed in different ways in different studies. In general, there is an overreliance on self-report, ranging from well-validated questionnaires to individual experimenter-made items [1, 20]. Additionally, some studies distinguish between the different types of abuse and their respective contributions, while others considered abuse in general or focused on one type without accounting for whether other types could occur simultaneously [1, 20, 21]. Others addressed different forms of childhood adversity, such as antipathy and neglect, alongside with abuse. While the literature on abuse is extensive, it is also abundant with mixed findings, spurring several recent umbrella reviews (see [9, 20, 22, 23]).

Assessing specific types of abuse is important because each may be predictive of different short- and long-term outcomes. For example, psychological abuse is often found to be strongly associated with depression later in life, compared to physical and sexual abuse [9, 24–26]. From a clinical perspective, victims of psychological abuse are identified and recommended for specific treatments, which usually address depressive symptoms in the context of the abuse or trauma they experienced. However, multiple types of abuse are likely to cooccur which can result in worse outcomes compared to the exposure to one type of abuse [27, 28]. Thus, individuals exposed to multiple forms of abuse require a custom-tailored treatment approach. In practice, however, such targeted evidence-based treatments may not always be available or have yet to be developed or refined.

Another challenge is elucidating the nature of the relationship between abuse and depression, with no single method that can conclusively establish a causal link. Much of the research has been cross-sectional and retrospective. For example, adults who report experiences of any type of abuse are at greater risk of mental health problems, including depression, compared to those without a history of abuse [29, 30]. In these studies, the reverse relationship, where depression predisposes individuals to abuse, is also possible. For example, the nature of the family environment during childhood and how the individuals are treated (e.g., neglect) could increase the risk for both abuse and depression. Powers et al. [24] found that, in addition to psychological abuse, neglect was a stronger predictor of adult depression compared to both physical and sexual abuse during childhood. Meta-analyses have also established that there is a consistent relationship between multiple forms of maltreatment, including psychological abuse, antipathy and neglect, and depression [9].

Furthermore, retrospective reports are subject to recall bias, known as mood-congruent recall, where individuals who are currently depressed could be more likely to remember negative events from their childhood [31–33]. This can skew research results and help explain why some meta-analyses report a stronger association between abuse and depression with clinical samples compared to other types of samples. Clinical samples may exclude individuals with a history of abuse but who are, nonetheless, well-adjusted [20, 34]. A more recent meta-analysis by Infurna et al. [9] also showed a stronger link between abuse and depression in clinical vs. nonclinical samples; however, their clinical category included data from high-risk community samples. In these types of samples, false negative rather than false positive reporting may be more common for traits that involve social stigma, including history of abuse [35]. In addition, mood-congruent recall in individuals with depression could allow them to remember both negative and positive events equally, whereas nondepressed control participants tend to remember positive events [36, 37].

Longitudinal studies can begin to establish a probable causal link, though it is not conclusive. Kendler and Aggen [37] conducted a study on the impact of sexual abuse in female twins that controlled for potential confounds related to the parents of the participants, including their socioeconomic status, depression history, and warmth and whether each twin lived with both biological parents up to a certain age. The authors also accounted for mood-congruent recall bias and measured depression at two time points via interview. A statistical model that did not include any of these confounds found that sexual abuse accounted for 9.6% of the variance observed in depression. However, after including these confounds, this estimate decreased to 4.4%, with current depression symptoms remaining a significant predictor of the recall of abuse-related experiences. It is important to highlight that, despite the seemingly low estimate, the odds ratio between child sexual abuse and depression was 1.83 (where an odds ratio of 1 indicates no relationship). Therefore, depending on how this ratio is interpreted, a low to moderate relationship remained. These results also suggest that more than half of the association between childhood sexual abuse and depression is not causal [37]. The limitations of this study include that all twins were white females born in Virginia; therefore, they are likely unrepresentative of the general population. In addition, although each twin was asked about the experience of the other twin, their reports often did not match, with rates of sexual abuse based on cotwin reports lower than those based on self-report. However, more than two-thirds of the twins reported that they did not tell anyone about their abuse experience, which could account for at least some of the inconsistency [37].

## 3. Abuse, Stress Reactivity, and Depression

There is a general consensus that childhood trauma is a significant vulnerability factor for depression and that both the chronicity [38] and the severity of depression in adulthood is related to the severity of childhood abuse [39]. How may childhood abuse increase the risk for depression in adulthood? It is well known that strong, frequent, and prolonged stress [40] can increase the risk of lifelong adverse mental health issues, including depression [41]. From a biological perspective, traumatic life experience, such as abuse, alter the physiology and even the structure of the brain [42], including long-term changes in neural networks involved in the regulation of the physiological response to stress [43, 44]. The response to threat is characterized by varying degrees of sympathetic (fight and flight) and parasympathetic (flag and faint) nervous system activation, depending on the event and the person. These experiences can sensitize the hypothalamic-pituitary-adrenal (HPA) axis and other systems involved in the stress response, so that those who are subjected to abuse become overly reactive to any environmental trigger or stressor [45, 46]. Consistent with this, childhood abuse is also a risk factor for a variety of stress-related physical health conditions, such as heart disease and diabetes [47].

It is important to highlight that genetic factors also influence the likelihood of developing depression when subjected to childhood abuse. Bradley et al. [48] found that polymorphisms in the gene for a particular corticotropin-releasing hormone (CRH) receptor influence (i.e., moderate) the strength of the relationship between childhood abuse and depression symptoms in adults. CRH, a key component of the HPA axis, is released by the hypothalamus and regulates the release of adrenocorticotropic hormone from the anterior pituitary gland. This hormone, in return, stimulates the release of stress-related steroid hormones (e.g., cortisol in humans), from the adrenal glands. Such physiological changes are also associated with emotional suffering and can leave individuals vulnerable to unhealthy methods of regulating stress, which contribute to the severity of depression and other types of psychopathology [49, 50]. These long-term changes in stress reactivity can also be expected to increase the risk for a variety of physical conditions, which research has confirmed. For example, childhood sexual abuse is associated with a variety of health problems, including immune issues and gastrointestinal and cardiopulmonary malfunction [46, 51]. Animal (i.e., preclinical) models can also add to our understanding of these changes, as well as establish cause and effect [52, 53].

There is also evidence that females are more vulnerable to long-term changes in stress reactivity compared to males, which may contribute to the increased prevalence of depression in women [54]. Women are twice as likely to be diagnosed with depression, compared to men, and are also more likely to have experienced some form of abuse (physical, emotional, or sexual) as a child [55]. In a study that examined the relationship between objective and subjective definitions of physical abuse, and the prevalence of depression, the proportion of women who experienced depression during their lifetime was the highest among those who were identified as victims of abuse [56, 57]. This increased risk, however, is not specific to depression since women who have experienced abuse or other types of trauma are more likely to develop a variety of other mental health conditions [58]. In addition, males and females respond to sexual abuse differently, where men tend to externalize their negative emotions, which can explain the high prevalence of aggressiveness and/or antisocial behavior, in contrast to that of depression in women who internalize their negative emotions [59].

Besides gender, the age at onset, as well as the extent, nature, and duration of abuse, can also affect depression [60, 61]. The social isolation of the victim, the identity of the abuser, and, importantly, the provision of needed support also influence the severity of depression [61]. Lack of support can lead to the repetition of the abuse, which also influences depression [63]. Despite the identity of the abuser, whether a family member, a friend, a caregiver, an authority figure, or a stranger, it still has severe negative consequences on the victims' emotional well-being and their perception of those around them [55]. In particular, traumatic experiences that occur during early life, such as abuse, have a dramatic impact on the development of schemas related to the perception of one's self, others, and the world [64]. In clinical practice, over 70% of clients report trauma because of abuse, which has reformed their cognitive schemas, including their pattern of thoughts, feelings, and behaviors compared to those who had no history of any abuse or trauma [65]. Both psychological and physical abuse adversely affect self-esteem and self-worth [66]. This trauma can also affect social relationships, with the individual feeling unworthy, isolated, and becoming socially withdrawn [67].

## 4. Types of Abuse

Below, we discuss the development of depression in relation to physical, psychological, and sexual abuse.

### 4.1. Physical Abuse

Physical abuse occurs as part of a constellation of behaviors including authoritarian control, anxiety-provoking behavior, and physical hurt or discomfort that is perpetrated by any person close to the victim. A meta-analysis by Stoltenborgh et al. [68] of 168 studies, with a combined total of 9,643,299 participants, found the worldwide prevalence of childhood physical abuse to be 17.7%, with a substantial difference between studies based on the judgement of professionals relative to self-report studies (0.3% vs. 22.6%, respectively). The most prevalent forms of physical abuse involve both physical punishment and domestic violence, which also affect children in the family, even if they are not the direct target [69, 70]. Cultural factors appear to strongly influence the nature of physical abuse in most countries, depending on whether hitting, punching, kicking, or beating is socially and legally acceptable [71]. If societies endorse parents' power, they tend to justify physical abuse and may consider it acceptable [21]. When children are inadvertently harmed by their parents' actions, some consider this an act of abuse [72], while others require the harm to be intentional for it to be defined as abusive. One of the challenges of addressing potential child victims of abuse is that parents or other close relatives may perceive their actions as appropriate means of disciplining or guiding the behavior of their children [71]. Thus, even if these actions were harmful, the intent to abuse could be absent or difficult to establish. Some of the literature on child abuse explicitly includes violence of any kind against children at home, school, or other settings [69, 73].

In contrast to physical abuse, physical punishment of children is still acceptable in many countries and not generally considered as an adverse childhood experience [74]. Whereas such practice has not been persistently confronted by legal reform and/or public education, the few existing prevalence studies suggest that it remains common [75–77]. It is now well-established that this practice is unhealthy for children, with a strong association between physical punishment and negative developmental outcomes [15]. A large body of research has linked physical punishment with child and adolescent social, emotional, cognitive, developmental, and behavioral problems. In particular, corporal punishment is a significant factor in the development of violent behavior, along with other problems, such as substance abuse, both in childhood and in adulthood [78, 79]. A cross-sectional study by Afifi et al. [74] found that adults who reported being spanked as children had increased risk of attempted suicide, heavy drinking, and other drug use. This relationship was still statistically significant after adjusting for the experience of physical and emotional abuse. In contrast, the depressed affect was no longer significantly related to spanking after this adjustment [74]. The limitations of this study, however, include that it was based on self-report and retrospective in nature, with single yes or no items to assess depressed affect, moderate to heavy drinking, street drug use, and suicide attempt. As the authors point out, depressed affect is not the same as major depression [74].

A study by Taillieu and Brownridge [80] that examined college students found both corporal punishment and psychological aggression to be significant predictors of lower self-esteem, depression, and anxiety. However, the relationship with depression could be offset by a combination of other factors, such as parental warmth, responsiveness, support, and type and consistency of disciplinary style (e.g., punishing misbehavior vs. reinforcing desirable behavior). Thus, corporal punishment may increase the risk of a later depression if the family environment is adverse in other ways. In contrast, psychological aggression remained a significant predictor of both lower self-esteem and anxiety, even after accounting for these factors [80]. Although depression was assessed through a well-validated multi-item questionnaire, some of the same limitations as in the Afifi et al. [74] study still apply, such as data is cross-sectional and retrospective, without any temporal or causal interaction observations. Furthermore, single self-report and retrospective elements were focused on both the sensitivity to spanking and mental health effects. Depressive affect, for example, was analyzed using one item rather than a sequence of questions required to produce a depression scale. This was in addition to a sample that is likely not representative of the general population. More longitudinal studies are needed to elucidate the relationship between corporal punishment and depression, while accounting for a number of other variables, including risk and protective factors, such as those related to the family environment, which can moderate this relationship.

Like other types of abuse, physical abuse is correlated with depression and other types of psychopathology later in life [81]. A substantial association between abuse history and depression has been well-established, but as discussed earlier, a direct cause and effect relationship remains elusive. For example, many men and women who suffer from depression often reveal painful experiences related to physical abuse in their life [82]. Especially if the abuse occurs on a regular basis, it can lead to chronic and extreme stress, and as discussed earlier, stress related to abuse can negatively affect the development of both the nervous and immune systems. Other ways in which physical abuse can increase the risk of depression indirectly is by feelings of worthlessness, low self-esteem [83], and self-suffering, ultimately affecting the individual's ability to cope with stressful situations [84]. Thus, individuals going through physical suffering also experience emotional suffering, and physical suffering can intensify the negative emotions experienced in the long term [85]. To cope with these negative emotions, the person may internalize the pain or express it in undesirable and maladaptive outbursts [86]. This helps explain, as adults, maltreated children are at an increased risk of behavioral, physical, and mental health problems, including severe and more persistent depression [87–89].

### 4.2. Sexual Abuse

According to the American Psychological Association [90], sexual abuse refers to “an undesired sexual activity, with perpetrators using force, making threats or taking advantage of victims not able to give consent,” in which children and adults can be exploited for pleasure or gratification without consent [91]. Sexual abuse happens in different forms, such as indecent exposure, fondling or sucking of sexual body parts, and forced sexual intercourse, whether it is oral sex or involves penetration [92, 93]. Force in this case does not only refer to physical force, but includes manipulation, coercion, threats, and any situations where a person is unable to consent. It is difficult to summarize however the prevalence of specific forms of sexual victimization among adults because definitions and measurement approaches vary substantially across studies [94]. A meta-analysis by Stoltenborgh et al. [95] of 331 studies, with a combined total of 9,911,748 participants, found the worldwide prevalence of child sexual abuse to be 11.8%, with a substantial difference between studies based on the judgement of professionals relative to self-report studies (0.4% vs. 12.7%, respectively). One reliable finding is that women experience sexual victimization at a higher rate than men [96, 97]. Based on self-report studies, the prevalence of childhood sexual abuse is 18% for females compared to 7.6% for males [95]. This difference could be, in part, due to a reluctance of men to disclose sexual abuse for a variety of reasons, such as being perceived as weak [95]. In contrast, no gender differences were found in the worldwide prevalence of physical [98] or emotional abuse [99], although a study by Meng et al. [96] did find men were more likely to be physically abused as children compared to women.

The effects of sexual abuse on victims include guilt and self-blame, self-esteem difficulties, poor sleep, suicidal ideation, anxiety, and depression [100, 101]. Childhood sexual abuse is a well-established risk factor for adult depression [102, 103]. A recent meta-analysis of prospective studies found positive associations between abuse during childhood and adolescence, and adult depression [104]. Experiencing sexual assault, including attempted and/or completed rape, is uniquely pathogenic and associated with the elevated risk for mental health issues when compared with other traumatic events [105]. A recent meta-analysis confirmed that the mental health impact of sexual assault is both broad and targeted and increases the risk for most domains of psychopathology, including trauma and stressor-related disorders, anxiety, and depression [106]. A qualitative review of the prevalence of various mental disorders in adult survivors of sexual abuse found that 13-51% meet diagnostic criteria for depression, 23-44% experience suicidal ideation, and 2-19% attempt to suicide [107]. Although sexually abused adults were at higher risk for many mental health problems, the most prevalent issue was depression. Several studies show that physical, emotional, and sexual abuse are all related to an elevated risk of depression and anxiety disorders in adulthood [108]. Other studies found a relationship between the severity of abuse and neglect, and the development of depression [109] and anxiety symptoms as an adult [110]. Hence, the more severe the abuse and neglect are, the more likely for the individuals to show symptoms of depression and anxiety.

Sexual harassment is another form of abuse, which is documented to have an overall negative and lasting impact on the psychological function of victims. More than half of harassment survivors [111] report negative consequences on their well-being, including negative feelings, such as anger, anxiety, fear, sadness, and depression [112]. The psychological impact of sexual harassment is related to the type of harassment and the context in which it occurs. For example, both occupational and gender-based harassment have been characterized as chronic stressors because of their often-unpredictable onset and protracted duration [113]. Severe forms of sexual harassment where an individual in authority either demands sexual favors or implies that such favors are required to avoid negative, or receive positive, educational or occupational outcomes can also involve actual bodily and sexual threat and injury. Thus, some survivors of sexual harassment experience one or more traumas, whereas others experience highly distressing and upsetting events that do not include traumas [114]. Survivors of childhood sexual abuse experience an increased risk of negative feelings and psychological disorders, such as helplessness, anxiety, and depression.

### 4.3. Psychological Abuse

Defining psychological abuse, a term often used interchangeably with emotional abuse, is difficult. The consequences of psychological abuse, regardless of how it is defined, differ depending on the context and the age of the victim [23]. In general, like other types of abuse, it can be defined as an intentional behavior to communicate to the victim that they have no value (i.e., they are worthless or unwanted) or something is wrong with them [21]. Thus, emotional abuse includes acts that disturb the emotional health of the individual [115]. Such acts include restricting a person's movements (e.g., where they can go and who they can interact with), denigration, ridicule, threats and intimidation, discrimination, rejection, and other nonphysical forms of hostile treatment [116]. Emotional abuse can represent an ongoing pattern of behavior, occurring across many different situations, or be limited to or triggered by a specific situation. It is the least reported type of abuse, and its prevalence is difficult to estimate [21]. A meta-analysis by Stoltenborgh et al. [99] of 46 studies, with a combined total of 7,082,279 participants, found the worldwide prevalence of childhood emotional abuse to be 26.7%, with a substantial difference between studies based on the judgement of professionals relative to self-report studies (0.3% vs. 36.3%, respectively). Prior research sometimes included the failure of a caregiver to provide an appropriate and supportive environment as a part of emotional abuse [115]. Arguably, however, this can be considered neglect. Neglect refers to the failure of a parent or a caretaker to provide the necessities of life, such as nutrition, healthcare, education, emotional safety, and safe living conditions [117]. It is thus distinguished from circumstances of poverty in that it can only occur where reasonable resources are available to the family or caregiver. A meta-analysis of 16 independent samples, with a combined total of 59,655 participants, found the worldwide prevalence of childhood emotional neglect to be 18.4% [98].

The vast majority of studies on childhood abuse have focused on the impact of either sexual or physical abuse [118], linking both types of abuse to adult depression [119, 120]. However, studies that have examined the impact of multiple types of abuse have shown childhood emotional abuse to be more strongly related to depression compared to sexual and physical abuse [24, 26, 61]. Despite this, psychological abuse against children received less attention globally than other types of abuse. Psychological health issues caused by emotional abuse form a substantial portion of the global burden of disease. While some mental health consequences have been researched ([121], p. 47), others have only recently been given attention, including psychiatric disorders like major depression and suicidal behavior ([122], p.15). When emotional abuse is severe and ongoing, a victim may lose their entire sense of self, sometimes without a single mark or bruise. Instead, the wounds are invisible to others, hidden in the self-doubt, worthlessness, and self-hatred the victim feels. In fact, many victims say that the scars from emotional abuse last far longer and are much deeper than those from physical abuse [123].

Several negative emotions are commonly self-reported by victims of psychological abuse, including anger, fear, shame, and guilt, which are also common indicators of depression [124]. Empirical research dealing with the consequences of childhood emotional abuse on adult functioning is scarce. Yet, it is mostly related to low self-esteem, impaired interpersonal relationships, negative perception of the world, depressive moods, anxiety, suicidal tendencies, eating disorders, and overall psychiatric symptomatology ([65, 125, 126], p.75; [30, 127, 128]). Emotionally abused victims may adopt a negative self-image [129] and believe that they are not worthy of parental attention [130] due to the introjection of parents' harmful behavior such as criticism and insults [131]. Adverse childhood experiences can lead to maladaptive behaviors (e.g., suicidal ideation and related behaviors) through impairment of the self-concept ([132], p. 470). Research suggests that maltreated children and youth have lower self-esteem and are more likely to engage in a range of risky behaviors, including substance abuse [133].

As for verbal abuse, which involves the use of words to cause harm and belittle others, it is considered in many cultures as a normal act, especially if it is used as a form of discipline [134]. For instance, evidence suggests that many parents consider shouting at their children a common harmless act [7]. However, this may cause emotional trauma and result in a long-lasting harm. Ongoing, repeated verbal abuse by parents, caretakers, or someone in a position of authority can drastically affect self-esteem, raise anxiety and confusion, and contribute to clinical depression in vulnerable individuals [135]. Constant assault on an individual's autonomy and sense of identity can erode their confidence and self-worth. Thus, it is not unusual for victims of verbal abuse to become depressed, consistent with the correlation between verbal abuse and feeling powerless or helpless [136].

The painful experiences of abuse can also affect mood regulation ([128], p.140). Therefore, in addition to low self-esteem and the increase of negativity [133], these experiences can lead to less effective coping strategies [137], such as rumination and behavioral avoidance, both associated with emotional abuse during childhood and strongly related to depression [138]. Experiences of early emotional abuse may be particularly damaging since maltreatment is perpetrated directly by primary attachment figures, which could have greater potency in activating a negative model of the self and others [25]. Such experiences can also increase attachment insecurity, avoidance, and isolation, which also play a significant role in the development of depression [104]. As already mentioned, consistent emotional abuse takes its toll, leading to a breakdown in the ability to effectively resist or cope with these experiences ([132], p. 448). This is when individuals are more likely to become depressed; unable to escape anger, fear, shame, and guilt, they may attempt to cope by inhibiting all of these emotions. For example, a person who has difficulties revealing the concealed pain that resulted from abuse may also be unable to effectively understand and deal with their depression [139], with possible negative implications for treatment outcomes.

## 5. Comparing the Impact of Different Types of Abuse

Relatively few studies have addressed how different types of childhood abuse relate to developing depression in adulthood [140]. A recent meta-analysis by Nelson et al. [140] compared effect sizes for different types of childhood maltreatment to the mean across all types. The types included emotional, physical, and sexual abuse, as well as physical and emotional neglect. They found that childhood emotional neglect was the most common form of maltreatment in depression (43.86% vs. mean of 31.9%) but that emotional abuse was more strongly related to depression severity (a correlation of 0.30 vs. mean of 0.25). The risk of depression, however, did not depend on type, with a grand mean odds ratio of 3.01. The highest odds ratios were for emotional abuse and multiple forms of maltreatment (3.82 and 4.13, respectively), consistent with other studies that have implicated emotional (i.e., psychological) abuse in the risk for depression (e.g., [24, 141, 142]) as well as found a dose-response effect where multiple types of childhood adversity increase the risk for negative health outcomes [28].

A cross-sectional study by Hovens et al. [142] examined whether childhood emotional neglect, as well as psychological, physical, and sexual abuse, is related to current pure anxiety, pure depression, or comorbid anxiety and depression. The participants were 1931 adults from the Netherlands Study of Depression and Anxiety who reported on whether they experienced either neglect or abuse before the age of 16. The study found that both emotional neglect and all types of abuse increase the risk for psychopathology; therefore, there was no unique relationship between the type of adversity experienced and specific disorders. However, emotional neglect and psychological abuse had a stronger relationship with pure depression than pure anxiety [142]. After adjusting for age, gender, and education, the odds ratio for pure anxiety compared to no disorder was 2.03 for individuals who reported at least some psychological abuse. The odds ratio was 3.23 for pure depression compared to no disorder. Overall, the odds were highest for comorbid anxiety and depression [142]. A more recent cross-sectional study by Dye [141] compared 748 college students who self-reported childhood emotional, physical, or sexual abuse or combined physical and sexual abuse during childhood. The levels of depression, anxiety, and stress were higher for those who experienced emotional abuse compared to those who reported only physical, sexual, or both physical and sexual abuse. For example, the correlation between level of depression and emotional abuse was 0.387 compared to 0.212 and 0.170 for physical and sexual abuse, respectively [141]. Similar differences were observed between the correlations for anxiety and stress. Thus, like in Hovens et al. [142], emotional abuse was not a unique predictor of depression.

Another cross-sectional study by Meng et al. [96] compared the prevalence of depression and other mental disorders in a sample of 23,395 adults from the 2012 Canadian Community Health Survey who self-reported childhood physical and sexual abuse. As in the study of Hovens et al. [142], the abuse took place before the age of 16. Meng et al. [96] found increased risk of depression for both types of abuse. The odds ratios for physical and sexual abuse were comparable and ranged between 2 and 3. Once again, the increased risk was not specific to depression. The risk was also higher for other internalizing disorders, such as bipolar disorder and generalized anxiety disorder, as well as externalizing disorders, including alcohol and other drug use disorders.

A cross-sectional study by Spinazzola et al. [143] found evidence for somewhat distinct clinical profiles in victims of psychological maltreatment, including neglect and abuse, compared to those of physical and sexual abuse in a sample of 5616 children from the National Child Traumatic Stress Network. Specifically, psychological maltreatment was the strongest predictor of internalizing problems, including depression and anxiety, as well as attachment and self-esteem issues. In addition, it was also the strongest predictor of substance abuse [143]. Similarly, in a cross-sectional study of 378 adults, Powers et al. [24] found that both psychological abuse and emotional neglect were more strongly related to adult depression compared to both physical and sexual abuse. A study of 176 adolescents and emerging adults with depression by La Rocque et al. [144] also found evidence for a distinct, age-dependent effect of childhood emotional abuse, but not physical and sexual abuse. The adolescents with a history of emotional abuse reported less severe stressful life events prior to the onset of their depression compared to emerging adults, suggesting differences in response to stress between the two age groups [144].

Thus, in general, emotional abuse appears to be at least as, if not more, damaging when compared to other types of abuse. Nonetheless, other research that has assessed different types of abuse has found nonsexual forms of abuse (i.e., physical and emotional abuse) to have similar consequences and to include depression, anxiety, and behavioral problems, such as aggression [145]. Vachon et al. [145] studied 1191 maltreated and 1099 nonmaltreated children from low-income families, comparable in racial or ethnic diversity, and other demographic factors. Both physical and emotional abuse, as well as neglect, predicted similar and broad internalizing and externalizing problems, with sexual abuse not related to either [145]. The authors also point out that sexual abuse is less frequent and almost always accompanied by other types of abuse. This lack of variability has a side effect of greatly reducing the correlation between sexual and nonsexual maltreatment and making it very difficult to tease apart its consequences [145]. The authors argue that prevention and treatment strategies should focus on targeting underlying common factors, rather than being tailored to specific types of abuse. Furthermore, emotional abuse should not be dismissed as less important, as it causes comparable harm to physical abuse [145].

## 6. Conclusion

Exposure to abuse has been consistently shown to increase the odds of depression but also a variety of other mental health problems. Longitudinal research has established that the experience of physical, sexual, and psychological abuse during childhood or adolescence is a risk factor for the development of depression in adulthood. However, future research should focus on understanding the role of abuse in the development and maintenance of depression in clinical service to provide trauma-focused, cognitive-behaviorally based treatments that should serve as a first-line treatment for child and adult survivors, who are experiencing abuse- or trauma-related symptoms. Mental health practitioners working with survivors of any type of abuse should understand that it can lead to the development of both mental and physical health symptoms and offer evidence-based treatment, including trauma-focused treatments as appropriate. The mental health practitioner should also be aware of the fact that many sexual victimization survivors have multiple health concerns. There is a need for more research on the impact of abuse history on response to treatment for issues common among survivors. It is also vital to explore the history of abuse in referred cases to understand the underlying dynamics of their symptomology. In order to provide effective treatment to people with depression through psychological interventions, it is important to specifically target the underlying factors associated with abuse at any stage of their life. Different childhood adversities, with a particular emphasis on psychological, physical, or sexual types of abuse may influence the specific etiological pathways in depression, and understanding these pathways is beneficial for developing individually tailored interventions. Relatively few studies, however, have addressed the specific mechanisms through which each type of abuse can increase the vulnerability for depression later in life, with a general lack of longitudinal research. Thus, the implementation of custom-tailored treatments in clinical practice remains very difficult.

## References

[1] Mathews B., Pacella R., Dunne M. P., Simunovic M., Marston C. (2020). Improving measurement of child abuse and neglect: a systematic review and analysis of national prevalence studies. *PLoS One*.

[2] Naughton A. M., Maguire S. A., Mann M. K. (2013). Emotional, behavioral, and developmental features indicative of neglect or emotional abuse in preschool children: a systematic review. *JAMA Pediatrics*.

[3] McFarlane A., Clark C. R., Bryant R. A., Williams L. M., Niaura R., Paul R. H. (2005). The impact of early life stress on psychophysiological, personality and behavioral measures in 740 non-clinical subjects. *Journal of Integrative Neuroscience*.

[4] Radell M. L., Abo Hamza E., Moustafa A. A. (2020). Depression in post-traumatic stress disorder. *Reviews in the Neurosciences*.

[5] Soriano K. (2020). *Depression central: tell me all i need to know about depression*.

[6] Shelton J. (2019). *Depression definition and DSM-5 diagnostic criteria*.

[7] Christ C., de Waal M. M., Dekker J. J. M. (2019). Linking childhood emotional abuse and depressive symptoms: the role of emotion dysregulation and interpersonal problems. *PLoS One*.

[8] Lopizzo N., Chiavetto L. B., Cattane N. (2015). Gene-Environment interaction in major depression: focus on experience-dependent biological systems. *Frontiers in Psychiatry*.

[9] Infurna M. R., Reichl C., Parzer P., Schimmenti A., Bifulco A., Kaess M. (2016). Associations between depression and specific childhood experiences of abuse and neglect: a meta-analysis. *Journal of Affective Disorders*.

[10] Muscatell K. A., Slavich G. M., Monroe S. M., Gotlib I. H. (2009). Stressful life events, chronic difficulties, and the symptoms of clinical depression. *Journal of Nervous and Mental Disorders*.

[11] Lu W., Mueser K. T., Rosenberg S. D., Jankowski M. K. (2008). Correlates of adverse childhood experiences among adults with severe mood disorders. *Psychiatric Services*.

[12] Gallo A., Wertz C., Blavier A. (2016). When the child reveals a sexual abuse: the real-life experience of the couple, its functions and the consequences. *Neuropsychiatrie de l'Enfance et de l'Adolescence*.

[13] Xiang Y., Wang W., Guan F. (2018). The relationship between child maltreatment and dispositional envy and the mediating effect of self-esteem and social support in young adults. *Frontiers in Psychology*.

[14] Schulz P., Beblo T., Ribbert H., Kater L., Spannhorst S., Driessen M. (2017). How is childhood emotional abuse related to major depression in adulthood? The role of personality and emotion acceptance. *Child Abuse Neglect*.

[15] Durrant J., Ensom R. (2012). Physical punishment of children: lessons from 20 years of research. *Canadian Medical Association Journal*.

[16] Kirchberger I., Maleckar B., Meisinger C., Linseisen J., Schmauss M., Baumgärtner J. (2019). Long-term outcomes in patients with severe depression after in-hospital treatment - study protocol of the depression long-term Augsburg (DELTA) study. *BMJ Open*.

[17] Leary C. E., Kelley M. L., Morrow J., Mikulka P. J. (2008). Parental use of physical punishment as related to family environment, psychological well-being, and personality in undergraduates. *Journal of Family Violence*.

[18] Nanni V., Uher R., Danesei A. (2012). Childhood maltreatment predicts unfavorable course of illness and treatment outcome in depression: a meta-analysis. *The American Journal of Psychiatry*.

[19] Baker A. J. L. (2009). Adult recall of childhood psychological maltreatment: definitional strategies and challenges. *Children and Youth Services Review*.

[20] Maniglio R. (2010). Child sexual abuse in the etiology of depression: a systematic review of reviews. *Depression and Anxiety*.

[21] Leeb R. T., Lewis T., Zolotor A. J. (2011). A review of physical and mental health consequences of child abuse and neglect and implications for practice. *American Journal of Lifestyle Medicine*.

[22] Hailes H. P., Yu R., Danese A., Fazel S. (2019). Long-term outcomes of childhood sexual abuse: an umbrella review. *The Lancet Psychiatry*.

[23] Maniglio R. (2009). The impact of child sexual abuse on health: a systematic review of reviews. *Clinical Psychology Review*.

[24] Powers A., Ressler K. J., Bradley R. G. (2009). The protective role of friendship on the effects of childhood abuse and depression. *Depression and Anxiety*.

[25] Shapero B. G., Black S. K., Liu R. T. (2014). Stressful life events and depression symptoms: the effect of childhood emotional abuse on stress reactivity. *Journal of Clinical Psychology*.

[26] Spertus I. L., Yehuda R., Wong C. M., Halligan S., Seremetis S. V. (2003). Childhood emotional abuse and neglect as predictors of psychological and physical symptoms in women presenting to a primary care practice. *Child Abuse & Neglect*.

[27] Finkelhor D., Ormrod R. K., Turner H. A. (2007). Polyvictimization and trauma in a national longitudinal cohort. *Development and Psychopathology*.

[28] Hughes K., Bellis M. A., Hardcastle K. A. (2017). The effect of multiple adverse childhood experiences on health: a systematic review and meta-analysis. *The Lancet Public Health*.

[29] Hopfinger L., Berking M., Bockting C. L., Ebert D. D. (2016). Emotion regulation mediates the effect of childhood trauma on depression. *Journal of Affective Disorder*.

[30] Rich D. J., Gingerich K. J., Rosen L. A. (1997). Childhood emotional abuse and associated psychopathology in college students. *Journal of College Students Psychotherapy*.

[31] Lewinsohn P. M., Rosenbaum M. (1987). Recall of parental behavior by acute depressives, remitted depressives, and nondepressives. *Journal of Personality and Social Psychology*.

[32] Lewis P. A., Critchley H. D. (2003). Mood-dependent memory. *Trends in Cognitive Sciences*.

[33] Zetsche U., Bürkner P.-C., Renneberg B. (2019). Future expectations in clinical depression: biased or realistic?. *Journal of Abnormal Psychology*.

[34] Jumper S. A. (1995). A meta-analysis of the relationship of child sexual abuse to adult psychological adjustment. *Child Abuse & Neglect*.

[35] Moffitt T. E., Caspi A., Taylor A. (2010). How common are common mental disorders? Evidence that lifetime prevalence rates are doubled by prospective versus retrospective ascertainment. *Psychological Medicine*.

[36] Gilboa E., Roberts J. E., Gotlib I. H. (1997). The effects of induced and naturally occurring dysphoric mood on biases in self-evaluation and memory. *Cognition & Emotion*.

[37] Kendler K. S., Aggen S. H. (2014). Clarifying the causal relationship in women between childhood sexual abuse and lifetime major depression. *Psychological Medicine*.

[38] Johnstone J. M., Luty S. E., Carter J. D., Mulder R. T., Frampton C., Joyce P. R. (2009). Childhood neglect and abuse as predictors of antidepressant response in adult depression. *Depression and Anxiety*.

[39] Korkeila J., Vahtera J., Nabi H., Kivimäki M., Korkeila K., Sumanen M. (2010). Childhood adversities, adulthood life events and depression. *Journal of Affective Disorder*.

[40] Shonkoff J. P., Garner A. S. (2012). The life-long effects of early childhood adversity and toxic stress. *Pediatrics*.

[41] Gunnar M. R., Fisher P. A., Early Experience, Stress, and Prevention Network (2006). Bringing basic research on early experience and stress neurobiology to bear on preventive interventions for neglected and maltreated children. *Development and Psychopathology*.

[42] Opris, Loan, Casanova, Manuel F. (2017). *The physics of the mind and brain disorders: Integrated neural circuits supporting the emergence of mind*.

[43] Burns E. E., Jackson J. L., Harding H. G. (2010). Child maltreatment, emotion regulation, and posttraumatic stress: the impact of emotional abuse. *Journal of Aggressive Maltreatment Trauma*.

[44] Penza K. M., Heim C., Nemeroff C. B. (2003). Neurobiological effects of childhood abuse: implications for the pathophysiology of depression and anxiety. *Archives of Women's Mental Health*.

[45] Heim C., Newport D. J., Heit S. (2000). Pituitary-adrenal and autonomic responses to stress in women after sexual and physical abuse in childhood. *JAMA*.

[46] Sachs-Ericsson N., Cromer K., Hernandez A., Kendall-Tackett K. (2009). A review of childhood abuse, health, and pain-related problems: the role of psychiatric disorders and current life stress. *Journal of Trauma & Dissociation*.

[47] Jakubowski K. P., Cundiff J. M., Matthews K. A. (2018). Cumulative childhood adversity and adult cardiometabolic disease: a meta-analysis. *Health Psychology*.

[48] Bradley R. G., Binder E. B., Epstein M. P. (2008). Influence of child abuse on adult depression: moderation by the corticotropin-releasing hormone receptor gene. *Archives of General Psychiatry*.

[49] Klein D. N., Glenn C. R., Kosty D. B., Seeley J. R., Rohde P., Lewinsohn P. M. (2013). Predictors of first lifetime onset of major depressive disorder in young adulthood. *Journal of Abnormal Psychology*.

[50] Read J., Fosse R., Moskowitz A., Perry B. (2014). The traumagenic neurodevelopmental model of psychosis revisited. *Neuropsychiatry*.

[51] Irish L., Kobayashi I., Delahanty D. L. (2010). Long-term physical health consequences of childhood sexual abuse: a meta-analytic review. *Journal of Pediatric Psychology*.

[52] Maestripieri D., Carroll K. A. (1998). Child abuse and neglect: usefulness of the animal data. *Psychological Bulletin*.

[53] Teicher M. H., Tomoda A., Andersen S. L. (2006). Neurobiological consequences of early stress and childhood maltreatment: are results from human and animal studies comparable?. *Annals of the New York Academy of Sciences*.

[54] Bale T. L., Epperson C. N. (2015). Sex differences and stress across the lifespan. *Nature Neuroscience*.

[55] Banyard V., Williams L. M., Siegel J. (2001). The long-term mental health consequences of child sexual abuse: an exploratory study of the impact of multiple traumas in a sample of women. *Journal of Traumatic Stress*.

[56] Carlin A. S., Kemper K., Ward N. G., Sowell H., Gustafson B., Stevens N. (1994). The effect of differences in objective and subjective definitions of childhood physical abuse on estimates of its incidence and relationship to psychop. *Journal of Child Abuse & Neglect*.

[57] Hayes L. (2018). *Understanding Child Abuse*.

[58] National Institute of Mental Health (2017). *Abuse, Trauma, and Mental Health*.

[59] Hill J. (2009). Developmental perspectives on adult depression. *Psychoanalytic Psychotherapy*.

[60] Hill J., Davis R., Byatt M., Burnside E., Rollinson L., Fear S. (2000). Childhood sexual abuse and affective symptoms in women: a general population study. *Psychological Medicine*.

[61] Martins C. M. S., Baes C. V. W., De-Carvalho Tofoli S. M., Juruena M. F. (2014). Emotional abuse in childhood is a differential factor for the development of depression in adults. *The Journal of Nervous and Mental Disease*.

[62] Philpot T. (2009). *Understanding Child Abuse: The Partners of Child Sex Offenders Tell their Stories*.

[63] Fullerton G. (2011). Creating advocates: the roles of satisfaction, trust and commitment. *Journal of Retailing and Consumer Services*.

[64] Beck A. T. (2008). The evolution of the cognitive model of depression and its neurobiological correlates. *American Journal of Psychiatry*.

[65] Gibb B. E., Wheeler R., Alloy L. B., Abramson L. Y. (2001). Emotional, physical, and sexual maltreatment in childhood versus adolescence and personality dysfunction in young adulthood. *Journal of Peers Disorder*.

[66] Widom C. S., Czaia S. J., Kozakowski S. S., Chauhan P. (2018). Does adult attachment style mediate the relationship between childhood maltreatment and mental and physical health outcomes?. *Journal of Child Abuse & Neglect*.

[67] Abela J. R., Taylor G. (2003). Specific vulnerability to depressive mood reactions in schoolchildren: the moderating role of self-esteem. *Journal of Clinical Child and Adolescent Psychology*.

[68] Stoltenborgh M., Bakermans-Kranenburg M. J., IJzendoorn M. H., Alink L. R. A. (2013). Cultural–geographical differences in the occurrence of child physical abuse? A meta-analysis of global prevalence. *International Journal of Psychology*.

[69] Shokair Z., Abo Hamza E. (2020). Family violence and its impact on children’s mental health during COVID-19 pandemic. *International Journal of Instructional Technology and Educational Studies (IJITES)*.

[70] Turner H. A., Muller P. A. (2004). Long-term effects of child corporal punishment on depressive symptoms in young adults. *Journal of Family Issues*.

[71] Petersen A. C., Joseph J., Feit M. (2014). *Committee on Child Maltreatment Research, Policy, and Practice for the Next Decade: Phase II; Board on Children, Youth, and Families; Committee on Law and Justice*.

[72] Jaffee S. R., Caspi A., Moffitt T. E., Polo-Tomas M., Price T. S., Taylor A. (2004). The limits of child effects: evidence for genetically mediated child effects on corporal punishment but not on physical maltreatment. *Developmental Psychology*.

[73] Shumba A. (2001). Epidemiology and etiology of reported cases of child physical abuse in Zimbabwean primary schools☆. *Child Abuse & Neglect*.

[74] Afifi T. O., Ford D., Gershoff E. T. (2017). Spanking and adult mental health impairment: the case for the designation of spanking as an adverse childhood experience. *Child Abuse & Neglect*.

[75] Finkelhor D., Turner H., Wormuth B. K., Vanderminden J., Hamby S. (2019). Corporal punishment: current rates from a national survey. *Journal of Child and Family Studies*.

[76] Gershoff E. T. (2017). School corporal punishment in global perspective: prevalence, outcomes, and efforts at intervention. *Psychology, Health & Medicine*.

[77] World Health Orgaization World report on violence and health.

[78] Bender H. L., Allen J. P., McElhaney K. B. (2007). Use of harsh physical discipline and developmental outcomes in adolescence. *Development and Psychopathology*.

[79] Knox M. (2010). On hitting children: a review of corporal punishment in the United States. *Journal of Pediatric Health Care*.

[80] Taillieu T. L., Brownridge D. A. (2013). Aggressive parental discipline experienced in childhood and internalizing problems in early adulthood. *Journal of Family Violence*.

[81] Lindert J., Von Ehrenstien O. S., Grashow R., Braehler E., Weisskopf M. G. (2014). Sexual and physical abuse in childhood is associated with depression and anxiety over the life course: systematic review and meta-analysis. *International Public Health Journal*.

[82] Chirichella-Besemer D., Motta R. W. (2008). Psychological maltreatment and its relationship with negative affect in men and women. *Journal of Emotional Abuse*.

[83] Arslan G. (2016). Psychological maltreatment, emotional and behavioral problems in adolescents: the mediating role of resilience and self-esteem. *Child Abuse & Neglect*.

[84] Çelik Ç. B., Odacı H. (2019). Does child abuse have an impact on self-esteem, depression, anxiety and stress conditions of individuals?. *International Journal of Social Psychiatry*.

[85] Afifi T. O., Brownridge D. A., Cox B. J., Sareen J. (2006). Physical punishment, childhood abuse and psychiatric disorders. *Child Abuse Neglect*.

[86] Sousa C., Mason W. A., Herrenkohl T. I., Prince D., Herrenkohl R. C., Russo M. J. (2018). Direct and indirect effects of child abuse and environmental stress: a lifecourse perspective on adversity and depressive symptoms. *American Journal of Orthopsychiatry*.

[87] Cicchetti D., Rogosch F. A. (1994). The toll of child maltreatment on the developing child: insights from developmental psychopathology. *Child and Adolescent Psychiatric Clinics of North America*.

[88] Mulvaney M. K., Mebert C. J. (2010). Stress appraisal and attitudes towards corporal punishment as intervening processes between corporal punishment and subsequent mental health. *Journal of Family Violence*.

[89] Kessler R. C., Bromet J. E. (2013). The epidemiology of depression across cultures. *Annual Revision Public Health*.

[90] American Psychological Association (2021). *Sexual abuse*.

[91] Mullers E. S., Dowling M. (2008). Mental health consequences of child sexual abuse. *British Journal of Nursing*.

[92] Flores R. J., Campro-Arias A., Stimpson J. P., Chalela C. M., Reyes-Ortiz C. A. (2017). The association between past sexual abuse and depression in older adults from Colombia. *Journal of Geriatric Psychiatry and Neurology*.

[93] Schaefer G. A., Mundt I. A., Ahlers C. J., Bahls C. (2012). Child sexual abuse and psychological impairment in victims: results of an online study initiated by victims. *Journal of Child Sexual Abuse: Research, Treatment, & Program Innovations for Victims, Survivors, & Offenders*.

[94] Tjaden P., Thoennes N. (2017). *Prevalence, incidence and consequences of violence against women: findings from the National Violence Against Women Survey*.

[95] Stoltenborgh M., Van Ijzendoorn M. H., Euser E. M., Bakermans-Kranenburg M. J. (2011). A global perspective on child sexual abuse: meta-analysis of prevalence around the world. *Child Maltreatment*.

[96] Meng X., D’Arcy C. (2016). Gender moderates the relationship between childhood abuse and internalizing and substance use disorders later in life: a cross-sectional analysis. *BMC Psychiatry*.

[97] Miranda J. K., de la Osa N., Granero R., Ezpeleta L. (2013). Maternal childhood abuse, intimate partner violence, and child psychopathology: the mediator role of mothers’ mental health. *Violence Against Women*.

[98] Stoltenborgh M., Bakermans-Kranenburg M. J., Van Ijzendoorn M. H. (2013). The neglect of child neglect: a meta-analytic review of the prevalence of neglect. *Social Psychiatry and Psychiatric Epidemiology*.

[99] Stoltenborgh M., Bakermans-Kranenburg M. J., Alink L. R., Van Ijzendoorn M. H. (2012). The universality of childhood emotional abuse: a meta-analysis of worldwide prevalence. *Journal of Aggression, Maltreatment & Trauma*.

[100] Chen L. P., Murad M. H., Paras M. L. (2010). Sexual abuse and lifetime diagnosis of psychiatric disorders: systematic review and meta-analysis. *Mayo Clinical Proceeding*.

[101] Papalia N., Ogloff J. R. P., Cutajar M., Mullen P. E. (2018). Child sexual abuse and criminal offending: gender-specific effects and the role of abuse characteristics and other adverse outcomes. *Journal of Child Maltreatment*.

[102] Cutajar M. C., Mullen P. E., Ogloff J., Thomas S. D., Wells D. L., Spataro J. (2010). Psychopathology in a large cohort of sexually abused children followed up to 43 years. *Child Abuse & Neglect*.

[103] Ullman S. E., Vasquez A. L. (2015). Mediators of sexual revictimization risk in adult sexual assault victims. *Journal of Child Sexual Abuse*.

[104] Li M., D’Arcy C., Meng X. (2016). Maltreatment in childhood substantially increases the risk of adult depression and anxiety in prospective cohort studies: systematic review, meta-analysis, and proportional attributable fractions. *Psychological Medicine*.

[105] Chivers-Wilson K. A. (2006). Sexual assault and posttraumatic stress disorder: a review of the biological, psychological and sociological factors and treatments. *McGill Journal of Medicine*.

[106] Dworkin E. R., Pittenger S. L., Allen N. E. (2017). Disclosing sexual assault within social networks: a mixed-method investigation. *American Journal of Community Psychology*.

[107] Xu Y., Olfson M., Villegas L. (2013). A characterization of adult victims of sexual violence: results from the National Epidemiological Survey for Alcohol and Related Conditions. *Psychiatry*.

[108] Gibb B. E., Butler A. C., Beck J. S. (2003). Childhood abuse, depression, and anxiety in adult psychiatric outpatients. *Depression and Anxiety*.

[109] Teicher M. H., Samson J. A., Polaris A., McGreenery C. E. (2006). Sticks, stones, and hurtful words: relative effects of various forms of childhood maltreatment. *American Journal of Psychiatry*.

[110] Glover D. A., Loeb T. B., Carmona J. V., Sciolla A., Zhang M., Myers H. F. (2010). Childhood sexual abuse severity and disclosure predict posttraumatic stress symptoms and biomarkers in ethnic minority women. *Journal of Trauma & Dissociation*.

[111] Willness C. R., Steel P., Lee K. (2007). A meta-analysis of the antecedents and consequences of workplace sexual harassment. *Personnel Psychology*.

[112] Eom E., Restaino S., Perkins A. M., Neveln N., Harrington J. W. (2015). Sexual harassment in middle and high school children and effects on physical and mental health. *Clinical Pediatric*.

[113] Bucchianeri M. M., Eisenberg M. E., Wall M. M., Piran N., Neumark-Sztainer D. (2014). Multiple types of harassment: associations with emotional well-being and unhealthy behaviors in adolescents. *Journal of Adolescent Health*.

[114] Chiodo D., Wolfe D. A., Crooks C., Hughes R., Jaffe P. (2009). Impact of sexual harassment victimization by peers on subsequent adolescent victimization and adjustment: a longitudinal study. *Journal of Adolescent Health*.

[115] Crow T., Cross D., Powers A., Bradley B. (2014). Emotion dysregulation as a mediator between childhood emotional abuse and current depression in a low-income African-American sample. *Child Abuse & Neglect.*.

[116] Lindert J., de Luna J., Torres-Gonzales F. (2013). Abuse and neglect of older persons in seven cities in seven countries in Europe: a cross-sectional community study. *International Journal of Public Health*.

[117] Neumann E. (2017). Recollections of emotional abuse and neglect in childhood as risk factors for depressive disorders and the need for psychotherapy in adult life. *Journal of Nerve and Mental Disease*.

[118] Springer K. W., Sheridan J., Kuo D., Carnes M. (2007). Long-term physical and mental health consequences of childhood physical abuse: results from a large population-based sample of men and women. *Child Abuse & Neglect*.

[119] Egeland B. (2009). Taking stock: childhood emotional maltreatment and developmental psychopathology. *Child Abuse & Neglect*.

[120] Lansford J. E., Dodge K. A., Pettit G. S., Bates J. E., Crozier J., Kaplow J. A. (2002). A 12-year prospective study of the long-term effects of early child physical maltreatment on psychological, behavioral, and academic problems in adolescence. *Arch Pediatric Adolescent Medicine*.

[121] Alexander R. C., Levitt C. J., Smith W. L., Reece R. M., Ludwig S. (2001). Abusive head trauma. *Child Abuse: Medical Diagnosis and Management*.

[122] Bifulco A., Moran A. (1998). *Wednesday’s child: research into women’s experience of neglect and abuse in childhood, and adult depression*.

[123] Karakurt G., Silver K. E. (2013). Emotional abuse in intimate relationships: the role of gender and age. *Violence and Victims*.

[124] Lee S. W., Bae G. Y., Hyo-Deog R. (2018). Mediating effect of resilience on the association between emotional neglect and depressive symptoms. *Psychiatry Investigation*.

[125] Bifulco A., Moran P. M., Baines R., Bunn A., Stanford K. (2002). Exploring psychological abuse in childhood, II: association with other abuse and adult clinical depression. *Bull Menninger Clinical*.

[126] Hart S. N., Brassard M. R., Karlson H., Briere J., Berliner L., Bulkley J. A., Jenny C., Reid T. (1996). Psychological maltreatment. *The APSAC Handbook on Child Maltreatment*.

[127] Mazzeo S. E., Espelage D. L. (2002). Association between childhood physical and emotional abuse and disordered eating behaviors in female undergraduates: an investigation of the mediating role of alexithymia and depression. *Journal of Counseling Psychology*.

[128] Palmer S. E., Brown R. A., Rae-Grant N. I., Loughlin M. J. (2001). Survivors of childhood abuse: their reported experiences with professional help. *Social Work*.

[129] Frankel J. (2002). Exploring Ferenczi’s concept of identification with the aggressor: its role in trauma, everyday life and the therapeutic relationship. *Psychoanalyses Dialogues*.

[130] Chang J. J., Theodore A. D., Martin S. L., Runyan D. K. (2008). Psychological abuse between parents: associations with child maltreatment from a population-based sample. *Child Abuse & Neglect*.

[131] Doyle C. (2001). Surviving and coping with emotional abuse in childhood. *Clinical Child Psychiatry*.

[132] Harter S., Cicchetti D. (2015). I-self and me-self processes affecting developmental psychopathology and mental health. *Developmental Psychopathology, Theory and Method*.

[133] Oshri A., Carlson M. W., Kwon J. A., Zeichner A., Wickrama K. K. (2017). Developmental growth trajectories of self-esteem in adolescence: associations with child neglect and drug use and abuse in young adulthood. *Journal of Youth and Adolescence*.

[134] Vardigan B. (2019). *Yelling at children (verbal abuse). HealthDay*.

[135] Yabe Y., Hagiwara Y., Sekiguchi T. (2019). Parents’ own experience of verbal abuse is associated with their acceptance of abuse towards children from youth sports coaches. *The Tohoku Journal of Experimental Medicine*.

[136] De Bellis M. D., Nooner K. B., Scheid J. M., Cohen J. A. (2019). Depression in maltreated children and adolescents. *Child Adolescent Psychiatric Clinical North America*.

[137] Fergusson D. M., Boden J. M., Horwood L. J. (2008). Exposure to childhood sexual and physical abuse and adjustment in early adulthood. *Child Abuse Neglect*.

[138] O’Mahen H. A., Karl A., Moberly N., Fedock G. (2015). The association between childhood maltreatment and emotion regulation: two different mechanisms contributing to depression?. *Journal of Affective Disorders*.

[139] Jopling E., Tracy A., LeMoult J. (2020). Childhood maltreatment, negative self-referential processing, and depressive symptoms during stress. *Psychological Research and Behavior Management*.

[140] Nelson J., Klumparendt A., Doebler P., Ehring T. (2017). Childhood maltreatment and characteristics of adult depression: meta-analysis. *The British Journal of Psychiatry*.

[141] Dye H. L. (2020). Is emotional abuse as harmful as physical and/or sexual abuse?. *Journal of Child & Adolescent Trauma*.

[142] Hovens J. G. F., Wiersma J. E., Giltay E. J. (2010). Childhood life events and childhood trauma in adult patients with depressive, anxiety and comorbid disorders vs. controls. *Acta Psychiatrica Scandinavica*.

[143] Spinazzola J., Hodgdon H., Liang L. (2014). Unseen wounds: the contribution of psychological maltreatment to child and adolescent mental health and risk outcomes. *Psychological Trauma: Theory, Research, Practice, and Policy*.

[144] La Rocque C. L., Harkness K. L., Bagby R. M. (2014). The differential relation of childhood maltreatment to stress sensitization in adolescent and young adult depression. *Journal of Adolescence*.

[145] Vachon D. D., Kruger R. F., Rogosch F., Cicchetti D. (2015). Assessment of the harmful psychiatric and behavioral effects of different forms of child maltreatment. *JAMA Psychiatry*.

